# Supplementary material for: A Neanderthal/Denisovan GLI3 variant contributes to anatomical variations in mice
Source: Front Cell Dev Biol. 2023 Nov 2;11:1247361. doi: 10.3389/fcell.2023.1247361 (PMC10651735; doi:10.3389/fcell.2023.1247361)
Supplement: Supplementary file 3 [file Presentation1.pdf]

A Neanderthal/Denisovan GLI3 variant contributes to anatomical variations in mice

Ako Agata, Satoshi Ohtsuka, Ryota Noji, Hitoshi Gotoh, Katsuhiko Ono, Tadashi Nomura

Supplementary Figures (Figure S1-S4)

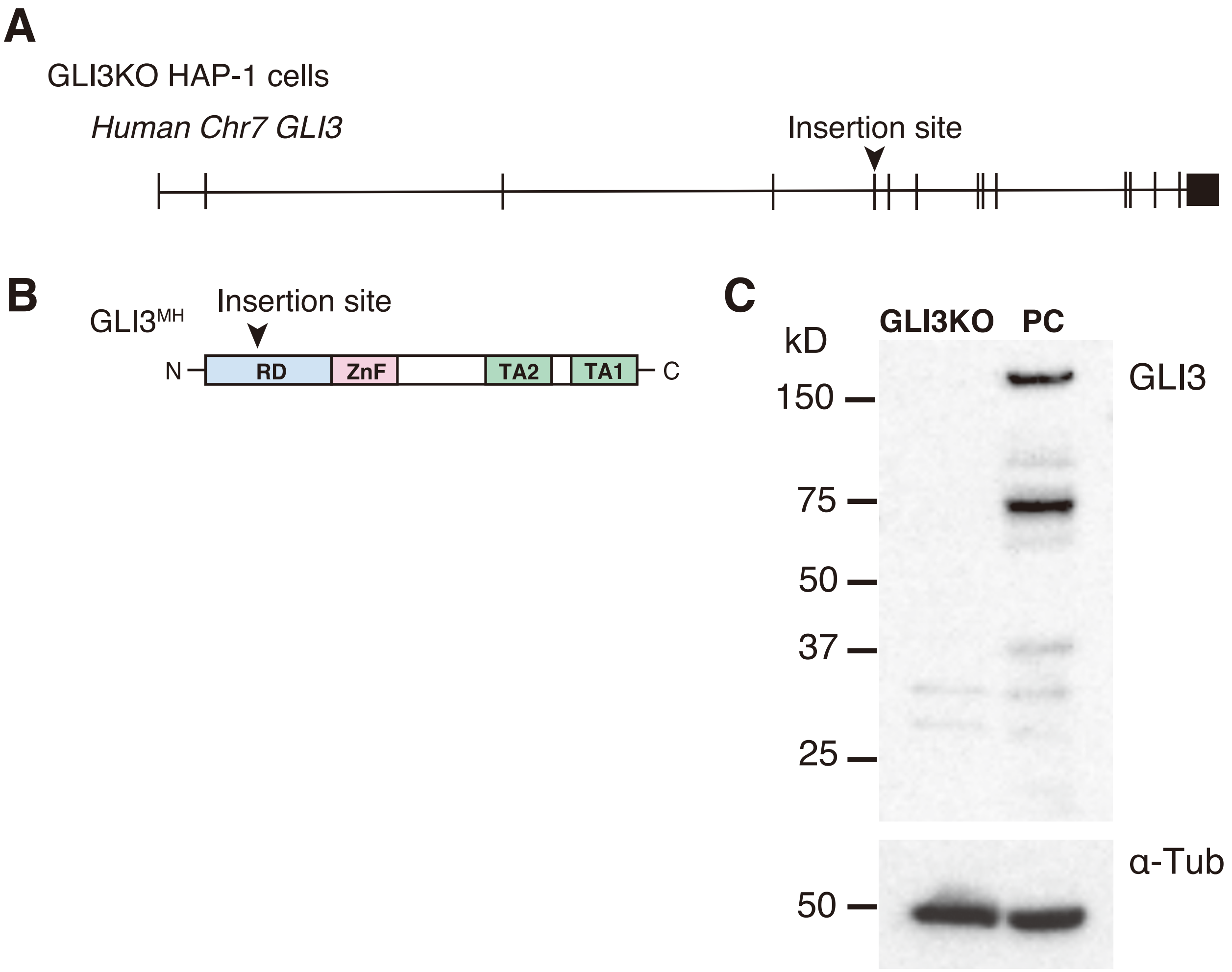

Figure S1. Generation of GLI3 knock out (KO) cell line. (A) Insertion site of *GLI3* genomic locus in HAP-1 cells. (B) The structure of human GLI3 and insertion site. (C) Western blotting of a GLI3 KO cell line and a parental cell line (PC; wild-type HAP-1 cells).

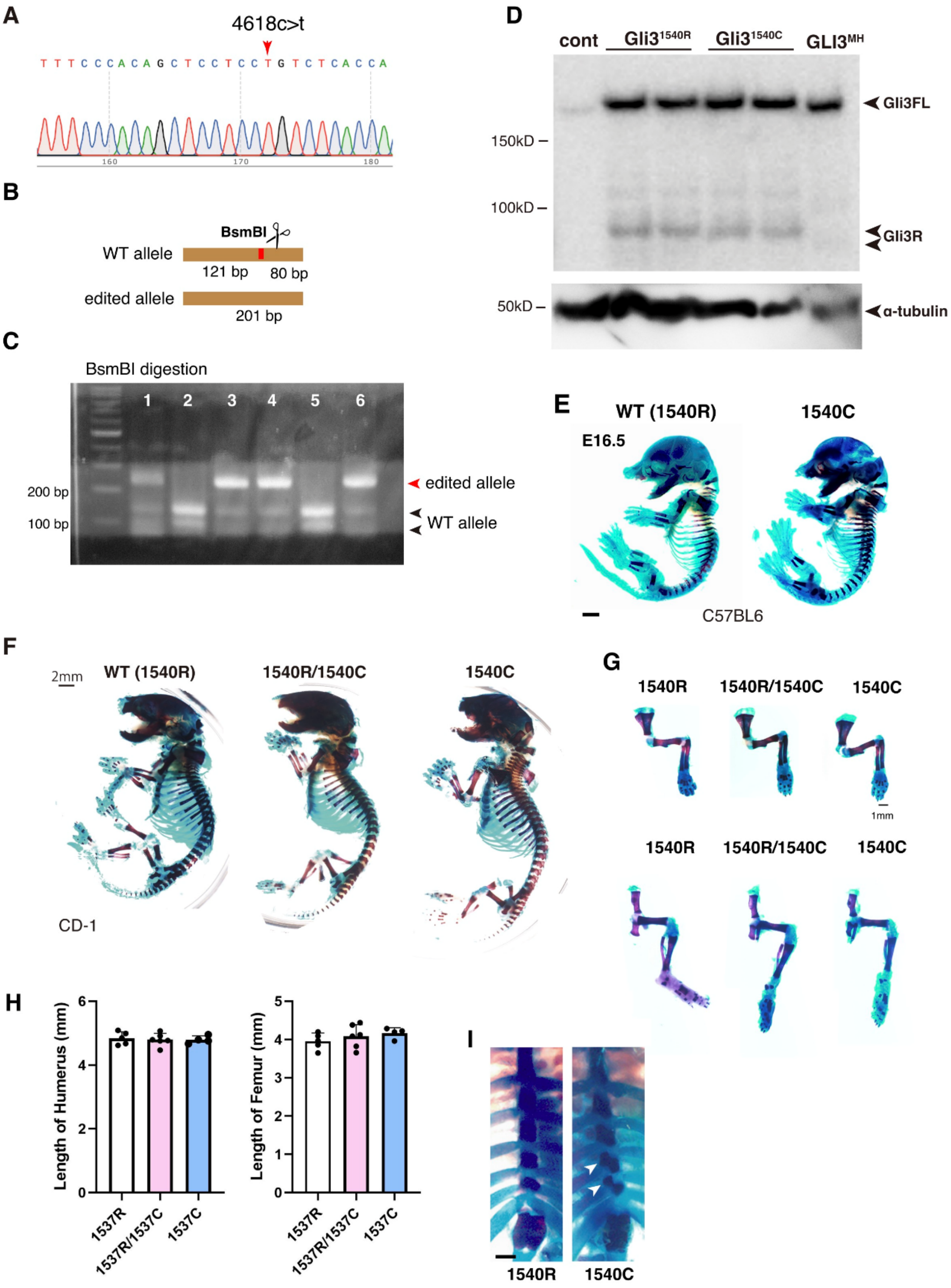

A
4618c>t
T T T C C C A C A G C T C C T C C T G T C T C A C C A
160
170
180
B
BsmBI
WT allele
121 bp
80 bp
edited allele
201 bp
C
BsmBI digestion
1
2
3
4
5
6
200 bp
100 bp
edited allele
WT allele
D
cont
Gli3 1540R
Gli3 1540C
GLI3 MH
Gli3FL
150kD
100kD
Gli3R
50kD
α-tubulin
E
WT (1540R)
1540C
E16.5
C57BL6
F
2mm
WT (1540R)
1540R/1540C
1540C
CD-1
G
1540R
1540R/1540C
1540C
1mm
1540R
1540R/1540C
1540C
H
Length of Humerus (mm)
1537R
1537R/1537C
1537C
Length of Femur (mm)
1537R
1537R/1537C
1537C
I
1540R
1540C

Figure S2. Validation of $Gli3^{R1540C}$ mouse genotype and macroscopic skeletal phenotypes. (A) Sequences of the PCR product from $Gli3^{R1540C}$ mouse. A red arrow indicates the nucleotide substitution corresponding to c4618t. (B) Restriction fragments of the PCR products digested by BsmBI. (C) Agarose gel electrophoresis showing restriction fragments. Black arrowheads indicate the fragment of the WT allele. A red arrowhead indicates the fragment of the edited allele. #1, 3, 4 and 6 indicate heterozygous. (D) Western blotting of HEK293T cells transfected with $Gli3^{1540R}$ or $Gli3^{1540C}$. HEK293T cells without transfection were used as a negative control (cont). Human GLI3 ($GLI3^{MH}$) were used as a positive control. (E, F) Whole mount skeletal images of WT, heterozygous (1540R/1540C) and homozygous (1540C/1540C) mice on C57BL6 (E) and CD-1 (F) backgrounds. (G) Fore and hind limbs in CD-1 mice. (H) Length of humerus and femur in CD-1 mice. (I) Crank-shaft sternum (white arrowheads) in homozygous mouse on CD-1 background. Scale bars: 2 mm (E, F) and 1 mm (G, I).

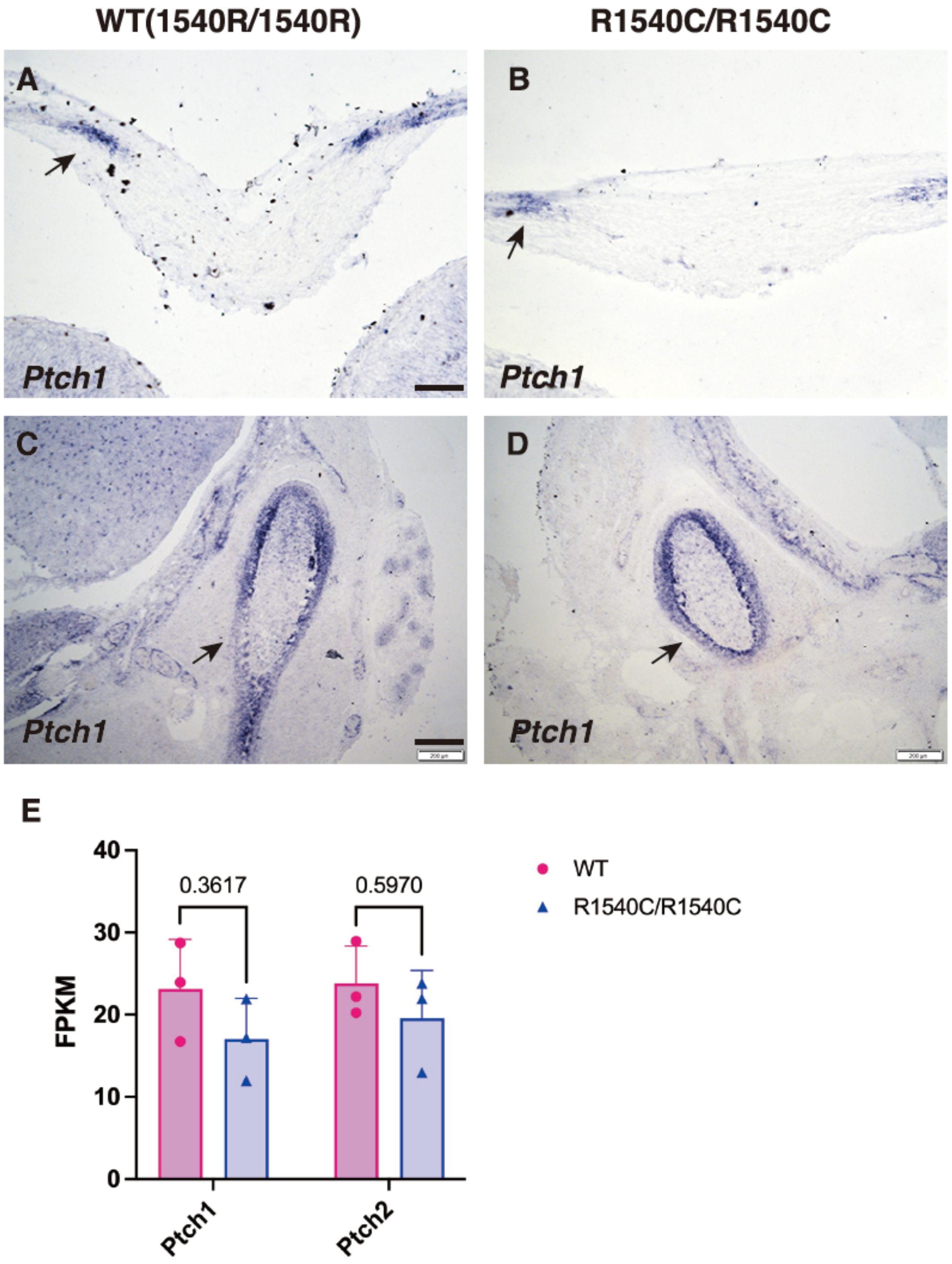

Figure S3. Expression of *Ptch1* in wild-type (WT) and GLI3^{R1540C} mice. (A-D) *In situ* hybridization of E17.5 WT (A, C) and GLI3^{R1540C} homozygous (B, D) mouse with *Ptch1* probe. Arrows indicate *Ptch1* expression in the developing frontal (A, B) and mandibular bones (C, D). (E) Expression levels (FPKM) of *Ptch1* in WT and GLI3^{R1540C} homozygous mouse. Statistical analysis was performed with Two-way ANOVA. Scale bars: 100 μm in A, 200 μm in C.

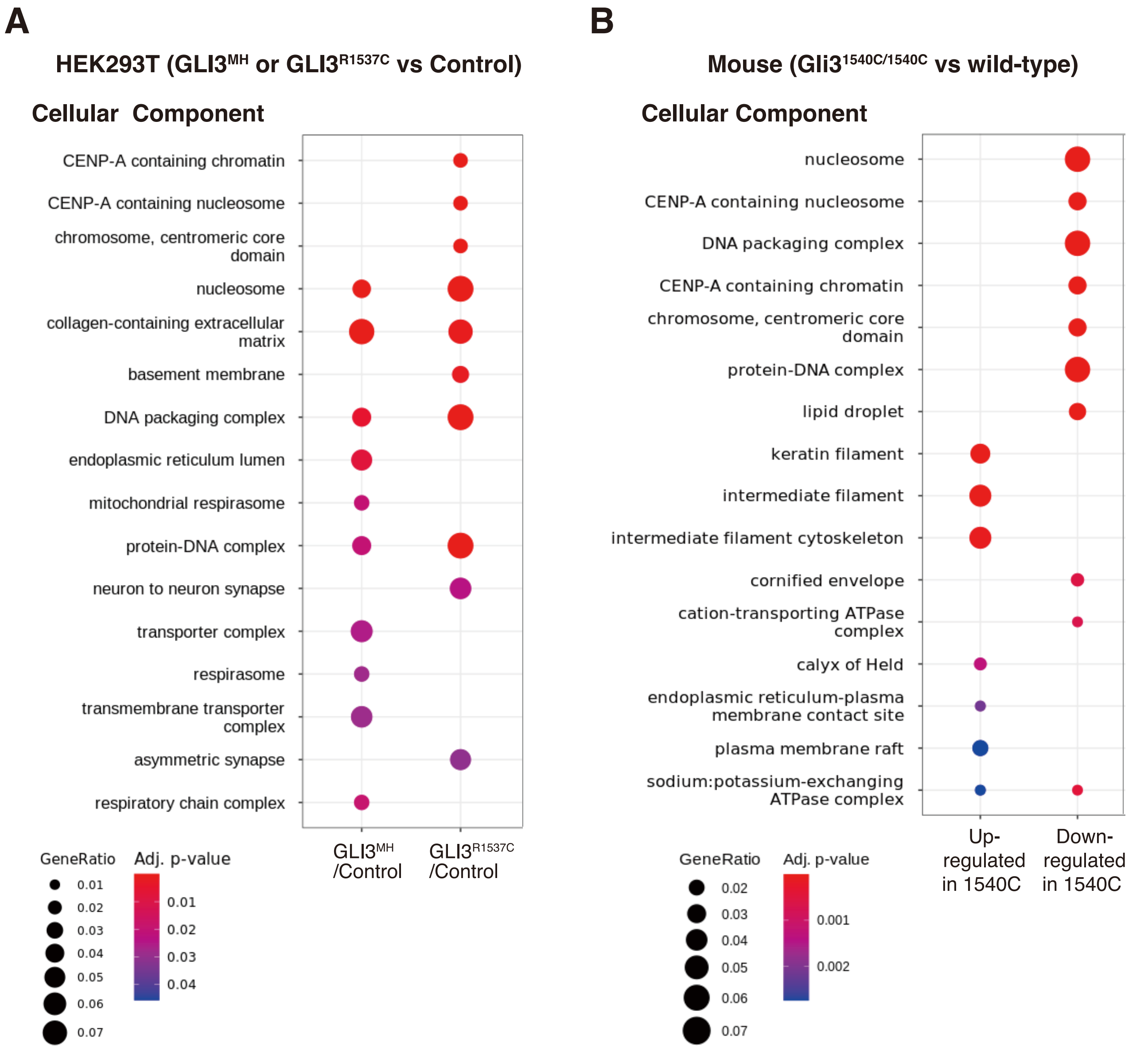

Figure S4 (A) GO enrichment analysis in comparisons of HEK293T cells overexpressing GLI3^{MH} or GLI3^{R1537C} versus control samples. Spots represent the top-ranking terms with gene ratios over 0.02. (B) GO enrichment analysis showing up/down-regulated terms in GLI3$^{1540C/1540C}$ mice compared with wild-type mice. Spots represent the top-ranking terms with gene ratios over 0.01.
